# Knowledge and perceptions about clinical research and its ethical conduct among college students from non-science background: a representative nation-wide survey from India

**DOI:** 10.1136/bmjph-2023-000748

**Published:** 2024-05-30

**Authors:** Jeffrey Pradeep Raj, Suraj Kallarakal Tomy, Amrutha Jose, Aadrika Kashyap, Joseph Varghese Kureethara, Tomy K Kallarakal

**Affiliations:** 1 Pharmacology, Kasturba Medical College, Manipal Manipal Academy of Higher Eduction, Manipal, Karnataka, India; 2 Pharmacology, Sri Ramachandra Medical College & Research Institute, Chennai, Tamil Nadu, India; 3 Department of Paediatrics, Manipal Hospitals, Bangalore, Karnataka, India; 4 National Institute of Immunohaematology, Mumbai, Maharashtra, India; 5 Seth GS Medical College and KEM Hospital, Mumbai, Maharashtra, India; 6 CHRIST (Deemed to be University), Bangalore, Karnataka, India

**Keywords:** COVID-19, Public Health, Community Health, ethics

## Abstract

**Introduction:**

Prior studies on public attitudes and awareness of clinical research (CR) in India before the pandemic identified significant knowledge gaps. Thus, we aim to investigate if the pandemic has influenced awareness and attitudes towards CR given the wide mass and social media coverage on drug/vaccine development process.

**Methods:**

This was an online survey conducted among young adults enrolled as students in a university in south India between 15 September 2022 and 31 October 2022. Until recently, these students attended online classes from various locations across India, likely reflecting a diverse regional and traditional background. Information regarding their sociodemographic status, awareness and perception about CR was collected and analysed applying the principle of descriptive and inferential statistics.

**Results:**

A total of N=4796 eligible survey responses were analysed. Approximately, 50% were unfamiliar with CR but 42.4% expressed willingness to participate in a future CR. The significant predictors for a higher favourable perception score were having heard about CR (p<0.001), known exactly what a CR means (p<0.001) and having known anyone who participated in a CR (p<0.001). The significant predictors for willingness to participate in a CR were those living in villages as against those living in cities (p=0.002), belongs to Brahm Govind (BG) Prasad’s socioeconomic status class 1 (p=0.023) or class 4 (p=0.011) as against those in class 5, those who have heard about CR (p=0.023), participated in CR (p<0.001), have known someone participated in a CR (p<0.001) or higher total score of favourable perceptions (p<0.001).

**Conclusions:**

While there was extensive media coverage on drug/vaccine development processes during the COVID-19 pandemic, significant knowledge gaps and concerns regarding various aspects of CR persisted. Although some individuals showed a positive view of CR and its societal benefits, the overall public awareness remains insufficient.

**WHAT IS ALREADY KNOWN ON THIS TOPIC**

- Public engagement in clinical research (CR) from India is hindered mainly by mistrust in, ignorance of, and misconceptions about CR.
- During the pandemic, media attention on CR was multifold.

**WHAT THIS STUDY ADDS**

- Post-COVID-19 pandemic, the changes in awareness and attitudes of general public towards the ethical conduct of CR in India.

**HOW THIS STUDY MIGHT AFFECT RESEARCH, PRACTICE OR POLICY**

- Despite wide media attention on the drug/vaccine development process during the pandemic, there were still notable gaps in knowledge regarding CR.
- The government may take up targeted educational initiatives and awareness campaigns to improve understanding and trust in CR among the lay public.

## Introduction

 India has become the key hub for conducting clinical research (CR),^1^ and its prowess in recruiting participants for clinical trials has established India as a pivotal location for health research. It is poised for significant growth, particularly due to its large and diverse population. As an emerging economy projected to lead globally, India’s commitment to independent medical research is crucial. The clinical trial industry and international partnerships are expected to flourish in this context.^2^ The diverse population in India, experiencing some diseases at rates comparable to other nations, adds to its advantages.^3^ Public engagement in CR is vital for ethical and methodological reasons.^4^ Amid the recent COVID-19 pandemic, media attention highlighted gaps in public awareness and participation, prompting this study. Despite regulatory advancements promoting CR and patient rights, gaps persist in public understanding,^5^ which in turn affects enrolment rates.^6^ The main obstacles to taking part in clinical trials have been identified as mistrust in, ignorance of, and misconceptions about CR.^7^ According to studies, raising knowledge affects people’s attitudes regarding clinical trials, enrolment and the advantages of taking part.^8^ Additionally, a well-informed populace is always better able to protect their rights. For the purpose of creating better awareness efforts, it is essential to comprehend the general public’s perspectives and understanding about CR.^9^ Studies that examined public attitudes and awareness of CR in India in the prepandemic era have reported several gaps in people’s knowledge on various aspects of CR.^10^ Only 42.5% (210/400) were aware about the term CR and 48.5% (194/400) needed permission from their family to participate. Besides this, there were significant number with concerns regarding voluntariness, perceived benefit to various stake holder, confidentiality and like-wise.^11^ Hence, we proposed to conduct this study primarily to determine the current awareness and attitudes towards CR after the COVID-19 pandemic given the wide media attention on the step-by-step development of COVID-19-related prevention and treatment strategies and public’s first-hand utilisation of new drugs and vaccines. The secondary objectives were to identify the predictors for a favourable perception, willingness to participate and to have already participated in a CR.

## Materials and methods

### Research question

The study was conducted to answer the question ‘What are the attitudes and awareness on CR among the general public of India represented by the young adult university-going students post-COVID-19 pandemic?’ We hypothesised that there would be some knowledge gaps still but the attitudes may have become more favourable.

### Study design and setting

This is a prospective cross-sectional study, conducted in India, where students were recruited from among those enrolled in the CHRIST (Deemed to be University) between 15 September 2022 and 31 October 2022. These students had recently started in-person classes and, until then, had been attending online classes from their homes ever since they were enrolled in the university. They had also not been staying outside their home state prior to this. Thus, their knowledge, perceptions and attitudes are likely to closely resemble their respective communities and geographical locations. Moreover, young adults enrolled from all over India in one university were considered as they are the ones who would be the future public once they pass out of their college studies in a short while. They would also be the earning members in the family soon and be participating in various decision-making

For this study, the standard definition of CR as recommended in the validated questionnaire was used after the initial introductory questions. The following was the definition used: *Clinical research is a scientific method of studying the effects, both positive and negative, of proposed new treatments [medications or devices] in human volunteers (healthy individuals or patients). Government authorities require convincing demonstration of benefit of a new treatment before giving approval to use it in the public at large. If adverse effects are found with a new treatment, then the benefits should outweigh the risk of adverse effects. Clinical trial, clinical study, and clinical research are all similar terms. Every medication in every pharmacy had to go through the clinical research process.*

### Eligibility criteria

All students from non-science majors (commerce, business administration and arts) studying in CHRIST (deemed to be University), Bengaluru were given an opportunity to participate in the survey. Students of all sexes above 18 years of age, who were willing to give digital written informed consent, were enrolled in this study. Students who were absent from the university on the day of the research team visiting their classroom were excluded. Students who did not complete filling the online form or failed to answer the trap question correctly were also excluded. The trap question was the same as one of the previous questions and if the responses to both these questions did not match, they were considered as not having answered the trap question correctly. Thus, the trap question helps to identify respondents who are not thoroughly reading the survey questions and are rushing through the survey by giving sub-optimal responses, thereby help maintaining the validity of the survey.^12^

### Sample size estimation and sampling strategy

Since the primary objective of the study was descriptive in nature, power-based sample size estimations were not done. A rule of thumb using the sample-to-variable ratio of 20:1 was considered (bare minimum being 5:1).^13^ Considering that our questionnaire consisted of approximately 50 questions, the sample size required for our survey would be 1000. However, we decided to include approximately 5000 students from the institute, so that there would be a good representation of all the 28 states and 8 union territories of India. Cluster random sampling was done with each classroom of approximately 60 students considered as a cluster. A list of classes and their courses were available from the academic section of the university and using a random number table, a 85 total of classrooms (clusters) was chosen and all students within the class were invited to participate in the study until the final sample size was reached.

### Study procedures, data sources and measurements

The research team visited the selected classrooms of the students and explained to them about the study. Subsequently, an online survey link made on Google Forms was circulated to the students of that particular class alone via their official emails. Once the students clicked the link, and gave their consent to participate, they were assessed for their eligibility and once confirmed, they gained access to the study questionnaire. If they were found ineligible, the Google Forms automatically considered them as screen failures and were excluded from the study. Care was taken that the link had access only to the students of that particular class and they were requested not to discuss with other students who are yet to be enrolled in order to prevent alterations in perceptions based on discussions with peers.

### Patient and public involvement

The development of the research question, study design, recruitment, reporting and dissemination plans in our research did not include input from patients or the general public. Nevertheless, individuals meeting the eligibility criteria were included in the study. While participants will not receive individual notifications of the study results, they can obtain them on request. Additionally, the results will be accessible through the scientific publication derived from the collected data.

### Variables

Data were collected under the following three domains

- Sociodemographic characteristics: age, sex, programme of study, socioeconomic status scale (based on BG Prasad scale modified for 2022^14^ geographical location based on permanent residential address and the type of residence location).
- Awareness: knowledge about CR, source of this knowledge, knowledge about others participation, willingness to participate and the likewise^7^
- Attitudes: respondents’ perception of how CR is conducted in India on various aspects such as confidentiality, voluntariness, government oversight, ensuring participant safety, compensation, the need and impact of CR.^7^

A score (ranging from 0 to 25) was derived based on the attitude questions where a score of one was given if the participant had a favourable attitude towards CR.

### Bias

Selection bias and external validity are a concern in survey studies. However, this is likely to have been mitigated in this study due to a sufficiently large sample size and that the respondents are more likely to represent all the various cultures and geographical regions of India. The internal validity was also ensured by means of the trap question to identify respondents who did not pay close attention to survey questions. Non-response bias has also been mitigated since the response rates were above 90%. Bias arising out of discussions among respondents was also mitigated by ensuring that students attempted the questionnaire only inside the classroom under the supervision of the research team and the respondents were also specifically requested not to discuss with other potential participants.

### Data management

The data were collected digitally using Google Forms. On study completion, the data were exported in the form of Microsoft Excel spreadsheets (Publisher: Microsoft Corporation, Redmond, Washington, 2016) and was stored in password-protected computers. Analyses were done only by study team members using the deidentified data. Data analysis was performed with SPSS for Windows, V.25.0 (Publisher: IBM, Armonk, New York, 2017).

### Quantitative variables and statistical analysis

Sociodemographic characteristics, knowledge and attitude questions were summarised using descriptive statistics. Categorical variables were expressed as frequency and percentage. Continuous variables were expressed as mean (SD). Normality was assessed using Kolmogorov Smirnov’s test. The predictors for favourable attitude scores, willingness to participate in future CR and having participated in a CR already were subjected to univariate and multivariable regression analysis; liner regression if the dependant variable was continuous (eg, Attitude scores) and binary logistic regression if the dependant variable was dichotomous (eg, willingness to participate of having already participated in a CR). Statistical significance was set at p<0.05.

## Results

### Demographic characteristics

A total of n=5536 students were approached for the study out of which n=5140 consented (92.8% response rate). The number finally included in the analysis was N=4796, the reasons for screen failure being students from science background (n=178) and those who did not respond to the questions pertaining to CR/failed to respond to the trap question correctly (n=166). The mean (SD) and median (Q1, Q3) age of the study participants was 19.69 (2.03) and 19 (18, 20), respectively, ranging from 18 to 34 years. The male respondents were slightly higher in number (52.9%) and a vast majority n=3875/4796; 80.8% doing an undergraduate course. The details of demographic characteristics and their geographic distribution from within India are summarised in table 1.

**Table 1:** Sociodemographic characteristics of the study participants

Variables	Category	Number(%)
Sex	Female	2258 (47.1)
	Male	2538 (52.9)
Locality	City	3621 (75.5)
	Town	886 (18.5)
	Village	289 (6.0)
Socio-economic class (BG Prasad Scale)	Class 1	3732 (77.8)
	Class 2	559 (11.7)
	Class 3	296 (6.2)
	Class 4	121 (2.5)
	Class 5	88 (1.8)
Programme of study	UG	3875 (80.8)
	PG	921 (19.2)
State/union territory	Andaman and Nicobar Island	3 (0.06)
	Andhra Pradesh	129 (2.69)
	Arunachal Pradesh	1 (0.02)
	Assam	85 (1.77)
	Bihar	103 (2.15)
	Chandigarh	14 (0.30)
	Chhattisgarh	48 (1)
	Dadra and Nagar Haveli and Daman and Diu	1 (0.02)
	Delhi	89 (1.86)
	Goa	19 (0.4)
	Gujarat	88 (1.83)
	Haryana	67 (1.4)
	Himachal Pradesh	11 (0.23)
	Jammu and Kashmir	11 (0.23)
	Jharkhand	104 (2.17)
	Karnataka	1251 (26.08)
	Kerala	897 (18.7)
	Lakshadweep	1 (0.02)
	Madhya Pradesh	94 (1.96)
	Maharashtra	129 (2.69)
	Manipur	1 (0.02)
	Meghalaya	3 (0.06)
	Nagaland	3 (0.06)
	Odisha	65 (1.36)
	Puducherry	16 (0.33)
	Punjab	112 (2.34)
	Rajasthan	195 (4.07)
	Sikkim	3 (0.06)
	Tamil Nadu	729 (15.2)
	Telangana	98 (2.04)
	Tripura	4 (0.08)
	Uttar Pradesh	232 (4.84)
	Uttarakhand	35 (0.73)
	West Bengal	155 (3.23)

### Awareness on CR

Almost half the study sample had not heard about CR (50.2%) before and a very small number had ever participated in a CR (2.4%). Only 42.4% (n=2032/4796) were willing to participate in CR in the future and 56.5% (n=2708/4796) stated that they would need to take permission from someone else before they can participate in a CR. The details of their knowledge/awareness regarding CR and their willingness to participate are summarised in table 2.

**Table 2:** Knowledge/awareness/willingness to participate in clinical research

	Category	Number (%)
Have you heard about clinical research?	No	2409 (50.2)
	Yes	2387 (49.8)
What was the source of information?^*^	Relatives	375 (7.8)
	Friends	859 (17.9)
	Colleagues	205 (4.3)
	Doctor	525 (10.9)
	Internet	1651 (34.4)
	Media	1244 (25.9)
	Other	425 (8.9)
Have you ever participated in clinical research?	No	4681 (97.6)
	Yes	115 (2.4)
Do you know what exactly a clinical research is?	No	3063 (63.9)
	Yes	1733 (36.1)
Do you know anyone who participated in clinical research?	No	4225 (88.1)
	Yes	571 (11.9)
How many people do you know who participated in clinical research?	1	270 (5.6)
	2	170 (3.5)
	3	45 (0.9)
	4	12 (0.3)
	5 or more	74 (1.5)
Do you know individuals who believe they were coerced to participate in clinical research?	No	4546 (94.8)
	Yes	250 (5.2)
Who were they coerced by?	Relatives	49 (1.0)
	Recruitment team	62 (1.3)
	Doctors	77 (1.6)
	Friends	64 (1.3)
Will you be willing to participate in clinical research?	No	2764 (57.6)
	Yes	2032 (42.4)
What kind of involvement in clinical research would you be willing to undertake?	Multiple days of stay in a confined unit (including overnight stay)	266 (5.5)
	Research done together with administration of standard medical care	492 (10.3)
	Multiple visits (each up to a few hours in duration)	371 (7.7)
	Single blood draw	756 (15.8)
	Single questionnaire (up to 20 min in duration)	1441 (30.0)
	Single visit (up to a few hours in duration with multiple interventions)	629 (13.1)
	Other	174 (3.6)
Would you have to take permission from someone else in order to participate in research?	No	2088 (43.5)
	Yes	2708 (56.5)
If you need to take permission to participate in clinical research, from who would it be?	Parents	2616 (54.5)
	Children	35 (0.7)
	Physician	684 (14.3)
	Friend	372 (7.8)
	Spouse	64 (1.3)
	Others	217 (4.5)

The definition and a brief idea on CR were given to the participants before this question was asked.

CRclinical research

Perceptions regarding their doctor’s care if not participating in CR were positive for 59.7% (n=2861/4796). Concerning confidentiality, 83.5% (n=4004/4796) valued it, but only 48.1% (n=2308/4796) believed it was safeguarded. Many were uncertain about trusting CR information from the pharmaceutical industry (47.2%, n=2263/4796) or academic institutions (44.9%, n=2154/4796). Doubt persisted on topics like adequate compensation for participation (62.1%, n=2978/4796) and adverse outcomes (56.4%, n=2703/4796). Questions arose on whether human participants in CR are treated like experimental animals (48.3%, n=2315/4796), receive sufficient information (43.0%, n=2063/4796) and if the government protects against unethical CR (44.0%, n=2111/4796). Uncertainty prevailed on making all CR results public (44.4%, n=2130/4796), media accuracy (43.6%, n=2089/4796), attributing harmful events in CR to experimental treatment (43.2%, n=2074/4796), hospitals participating in CR providing better healthcare (49.1%, n=2355/4796) and altruism as the sole valid reason for CR participation (45.9%, n=2201/4796).

### Attitudes regarding CR

Favourable perceptions included financial gain not being the primary motive for new treatments (47.9%, n=2297/4796), CR benefiting society (81.0%, n=3887/4796), CR as a crucial first step (80.3%, n=3851/4796), essential for developing treatments (50.6%, n=2428/4796), the advancement of science being the primary motive (69.6%, n=3338/4796), voluntary participation in CR (67.0%, n=3213/4796), doctors not compelling patients to join CR (52.0%, n=2492/4796), researchers ensuring CR safety (45.0%, n=2158/4796) and public involvement in CR (52.4%, n=2512/4796). Respondents perceived positive impacts on CR from collaborations with non-Indian industry partners (45.4%, n=2179/4796) and academic partners (36.5%, n=1752/4796). The complete details with regards to perceptions on CR are summarised in online supplemental table 1.

### Predictors of favourable perception

The significant predictors (adjusted β; SE; p value) for a higher favourable perception score (table 3) were having heard about CR (0.888, 0.152, p<0.001), known exactly what a CR means (1.593, 0.157, p<0.001), and having known anyone who participated in a CR (0.928, 0.234, p<0.001). The adjusted β value shows the predicted absolute increase in score from the mean score if that specific factor was present.

**Table 3:** Univariate and multivariable linear regression analysis for favourable perception score

Variables(N=4796)		Total score	Univariate analysis		Multivariable analysis	
		Mean±SD	β (SE)	P value	aβ (SE)	P value
Age in completed years (mean±SD)		–	−0.037 (0.036)	0.300	0.016 (0.057)	0.781
Sex	Male	11.05±5.039	−0.089 (0.146)	0.542	−0.133 (0.143)	0.353
	Female	11.14±5.055	Reference	–	Reference	–
Locality	Village	10.82±5.076	−0.333 (0.308)	0.281	−0.225 (0.304)	0.459
	Town	10.96±5.104	−0.186 (0.189)	0.327	−0.163 (0.186)	0.382
	City	11.15±5.030	Reference	–	Reference	–
Socioeconomic status class (BG Prasad scale modified for 2022)	Class 5	10.38±5.476	Reference	–	Reference	–
	Class 4	10.48±4.865	0.104 (0.707)	0.883	−0.088 (0.692)	0.899
	Class 3	11.09±5.229	0.720 (0.613)	0.240	0.782 (0.6)	0.193
	Class 2	10.92±5.004	0.550 (0.579)	0.342	0.616 (0.568)	0.278
	Class 1	11.16±5.033	0.783 (0.544)	0.151	0.765 (0.533)	0.152
Programme of study	PG	10.90±5.160	Reference	–	Reference	–
	UG	11.14±5.019	0.244 (0.185)	0.187	0.249 (0.292)	0.394
Heard about CR	No	10.36±5.326	Reference	–	Reference	–
	Yes	11.84±4.632	1.480 (0.144)	< 0.001	0.888 (0.152)	< 0.001
Know exactly about CR	No	10.38±5.245	Reference	–	Reference	–
	Yes	12.35±4.406	1.965 (0.149)	< 0.001	1.593 (0.157)	< 0.001
Participated in CR	No	11.06±5.035	Reference	–	Reference	–
	Yes	12.37±5.348	1.310 (0.476)	0.006	−0.061 (0.487)	0.901
Know anyone who participated in CR	No	10.91±5.041	Reference	–	Reference	–
	Yes	12.43±4.891	1.514 (0.224)	< 0.001	0.928 (0.234)	< 0.001

Adjusted R2 =0.00137.

CRclinical research

### Predictors of willingness to participate in a CR

The significant predictors (adjusted OR; 95% CI; p value) for willingness to participate in a CR (table 4) were those living in villages as against those living in cities (1.507; 1.169, 1.942; p=0.002), belongs to socioeconomic status (SES) class 4 (2.108; 1.150, 3.865; p=0.023) or class 1 (1.773; 1.095, 2.870; p=0.011) as against those in class 5, those who have heard about CR (1.160; 1.020, 1.318; p=0.023), know exactly what a CR means (0.774; 0.677, 0.884; p<0.001), participated in CR (3.097; 1.951, 4.917; p<0.001), have known someone participated in a CR (1.821; 1.497, 2.213; p<0.001) or higher total score of favourable perceptions (1.117; 1.102, 1.131; p<0.001).

**Table 4:** Univariate and multivariable logistic regression analysis for willingness to participate in clinical research (CR)

Variables(N=4796)		Willingness to participate in CR		Univariate analysis		Multivariable analysis	
		Yes (n=2032)	No (n=2764)	OR (95% CI)	P value	aOR (95% CI)	P value
		n (%)	n (%)				
Age in completed years (mean±SD)		19.63±1.998	19.74±2.050	0.974 (0.946 to 1.002)	**0.065**	0.980 (0.934 to 1.028)	0.408
Sex	Male	1093 (43.1)	1445 (56.9)	1.063 (0.947 to 1.192)	**0.301**	1.087 (0.963 to 1.227)	0.175
	Female	939 (41.6)	1319 (58.4)	Reference	–	Reference	–
Locality	Village	142 (49.1)	147 (50.9)	1.349 (1.061 to 1.715)	**0.014**	1.507 (1.169 to 1.942)	0.002
	Town	379 (42.8)	507 (57.2)	1.044 (0.900 to 1.211)	**0.571**	1.086 (0.927 to 1.271)	0.306
	City	1511 (41.7)	2110 (58.3)	Reference	–	Reference	–
Socioeconomic status class (BG Prasad scale modified for 2022)	Class 5	27 (30.7)	61 (69.3)	Reference	–	Reference	–
	Class 4	57 (47.1)	64 (52.9)	2.012 (1.130 to 3.582)	**0.016**	2.108 (1.150 to 3.865)	0.023
	Class 3	119 (40.2)	177 (59.8)	1.519 (0.913 to 2.527)	**0.146**	1.487 (0.871 to 2.539)	0.095
	Class 2	203 (36.3)	356 (63.7)	1.288 (0.793 to 2.092)	**0.300**	1.310 (0.786 to 2.181)	0.220
	Class 1	1626 (43.6)	2106 (56.4)	1.744 (1.104 to 2.757)	**0.020**	1.773 (1.095 to 2.870)	0.011
Programme of study	PG	369 (40.1)	552 (59.9)	Reference	–	Reference	–
	UG	1663 (42.9)	2212 (57.1)	1.125 (0.972 to 1.302)	**0.116**	1.033 (0.806 to 1.324)	0.799
Heard about CR	No	929 (38.6)	1480 (61.4)	Reference	–	Reference	–
	Yes	1103 (46.2)	1284 (53.8)	1.369 (1.220 to 1.535)	**<0.001**	1.160 (1.020 to 1.318)	0.023
Know exactly about CR	No	1274 (41.6)	1789 (58.4)	Reference	–	Reference	–
	Yes	758 (43.7)	975 (56.3)	1.092 (0.969 to 1.230)	**0.149**	0.774 (0.677 to 0.884)	< 0.001
Participated in CR	No	1945 (41.6)	2736 (58.4)	Reference	–	Reference	–
	Yes	87 (75.7)	28 (24.3)	4.371 (2.844 to 6.718)	**<0.001**	3.097 (1.951 to 4.917)	< 0.001
Know anyone who participated in CR	No	1687 (39.9)	2538 (60.1)	Reference	–	Reference	–
	Yes	345 (60.4)	226 (39.6)	2.297 (1.921 to 2.746)	**<0.001**	1.821 (1.497 to 2.213)	< 0.001
Total score (mean±SD)		12.60±4.632	9.99±5.053	1.116 (1.103 to 1.131)	**<0.001**	1.117 (1.102 to 1.131)	< 0.001

Nagelkerke R 2=0.122; Accuracy=63.2; Hosmer and Lemeshow test p value=0.196.

CRclinical research

### Predictors of those having participated in a CR already

The significant predictors (adjusted OR; 95% CIs; p value) for those having participated in a CR (table 5) were SES class 2 (0.243; 0.065, 0.914; p=0.036) as against SES class 5, those who have heard about CR (2.097; 1.233, 3.566; p=0.00 6), know exactly what a CR means (2.104; 1.361, 3.254; p=0.001) or have known someone participated in a CR (15.854; 10.290, 24.427; p<0.001).

**Table 5:** Univariate and multivariable logistic regression analysis for ever participated in clinical research (CR)

Variables(N=4796)		Participated in CR		Univariate analysis		Multivariable analysis	
		Yes (n=115)	No (n=4681)	OR (95% CI)	P value	aOR (95% CI)	P value
		n (%)	n (%)				
Age in completed years (mean±SD)		19.45±1.957	19.70±2.030	0.936 (0.846 to 1.036)	**0.201**	0.957 (0.809 to 1.131)	0.604
Sex	Male	54 (2.1)	2484 (97.9)	0.783 (0.540 to 1.134)	**0.196**	0.726 (0.488 to 1.082)	0.115
	Female	61 (2.7)	2197 (97.3)	Reference	–	Reference	–
Locality	Village	8 (2.8)	281 (97.2)	1.143 (0.549 to 2.381)	**0.721**	1.364 (0.618 to 3.031)	0.446
	Town	19 9 (2.1)	867 (97.9)	0.617 (0.880 to 0.533)	**0.617**	0.834 (0.489 to 1.423)	0.504
	City	88 (2.4)	3533 (97.6)	Reference	–	Reference	–
Socioeconomic status class (BG Prasad scale modified for 2022)	Class 5	4 (4.5)	84 (95.5)	Reference	–	Reference	–
	Class 4	5 (4.1)	116 (95.9)	0.905 (0.236 to 3.472)	**0.885**	0.616 (0.142 to 2.666)	0.517
	Class 3	6 (2.0)	290 (98.0)	0.434 (0.120 to 1.576)	**0.205**	0.357 (0.089 to 1.427)	0.145
	Class 2	8 (1.4)	551 (98.6)	0.305 (0.090 to 1.035)	**0.057**	0.243 (0.065 to 0.914)	0.036
	Class 1	92 (2.5)	3640 (97.5)	0.531 (0.191 to 1.478)	**0.225**	0.394 (0.128 to 1.214)	0.105
Programme of study	PG	19 (2.1)	902 (97.9)	Reference	–	Reference	–
	UG	96 (2.5)	3779 (97.5)	1.206 (0.733 to 1.983)	**0.461**	0.921 (0.403 to 2.103)	0.845
Heard about CR	No	20 (0.8)	2389 (99.2)	Reference	–	Reference	–
	Yes	95 (4.0)	2292 (96.0)	4.951 (3.047 to 8.046)	**<0.001**	2.097 (1.233 to 3.566)	0.006
Know exactly about CR	No	37 (1.2)	3026 (98.8)	Reference	–	Reference	–
	Yes	78 (4.5)	1655 (95.5)	3.854 (2.595 to 5.726)	**<0.001**	2.104 (1.361 to 3.254)	0.001
Know anyone who participated in CR	No	33 (0.8)	4192 (99.2)	Reference	–	Reference	–
	Yes	82 (14.4)	489 (85.6)	21.302 (14.070 to 32.251)	**<0.001**	15.854 (10.290 to 24.427)	<0.001
Total score (mean±SD)		12.37±5.348	11.06±5.035	1.055 (1.015 to 1.096)	**0.006**	0.997 (0.956 to 1.040)	0.891

Nagelkerke R 2=0.266; Accuracy=97.6; Hosmer and Lemeshow test p value=0.880.

CRclinical research

## Discussion

In a prospective, cross-sectional survey among N=4796 Indian university students from non-scientific backgrounds belonging to different states and union territories of India, conducted in September–October 2022, the study aimed to explore their knowledge and perceptions of CR. Roughly 50% were unfamiliar with CR, with only 2.4% having prior participation, but 42.4% expressed willingness to do so in the future. While participants recognised the voluntariness and societal benefits of CR, they lacked awareness about critical aspects like confidentiality, compensation, government’s role and safety. Predictors for a higher favourable perception score included prior awareness of CR, clear understanding regarding CR and acquaintance with previous CR participants. Willingness to participate was predicted by living in villages (as against a city), belonging to SES class 1 or 4 (as against class 5), prior CR awareness, understanding, personal participation, knowing someone who participated in previous CRs or a higher total favourable perception score. The significant predictors of participation were prior CR awareness, clear understanding regarding CR and knowing someone involved in CR.

Approximately, half the study sample had heard about CR but only a few had either participated in a CR or knew somebody who had participated in a CR. Almost one-quarter of the students reported that their knowledge on CR was from the media and the most common source reported in this study. This is in contrast to the study reported by Figer *et al* from Mumbai in the prepandemic era where the majority heard about CR from their doctors.^11^ This is most likely due to the media involvement in disseminating up to date information regarding the various stages of COVID-19 vaccine development in the country during the pandemic.^15^

We found that there were several knowledge gaps regarding the ethical conduct of CR in the country on important aspects like confidentiality, participant safety, compensation and government oversight. This is in stark contrast to what was reported from India in the prepandemic era, wherein respondents had positive perceptions on these aspects.^7 11^ This could be attributed to the mass and social media disseminating several unknowns about the novel coronavirus, many of which misinformed the public during the pandemic,^16^ for instance, an analysis of 45 scientific articles that analysed social media records from Twitter, Facebook, YouTube and Instagram identified three main themes, namely medical misinformation, vaccine development issues and controversies shrouding the pandemic.^16^ This even led to the WHO coin the term ‘infodemics’.^16^ This negative sentiment probably changed the public perceptions, thus reiterating the role mass and social media can play in creating awareness, thereby promoting public participation in CR. Misinformation and lack of trust in CR adversely affect healthcare utilisation of the general public, which in-turn have devastating consequence like it was seen during the COVID-19 pandemic, wherein those who were non-vaccinated for COVID-19 suffered from severe illness including death.^17^

With regard to the predictors of higher total favourable perception score, demographic characteristics such as age, sex, type of residence locality, SES class and programme of study had no significant influence on the total favourable perception scores. Instead, having heard of the term CR, a clear understanding of CR and having known somebody who participated in a CR seem to instil positive perceptions on CR and were also the factors associated with having participated in previous CR. In fact, those who knew somebody else participate in CR had approximately 15 times the higher odds for themselves having participated in a CR. These findings are similar to those reported by other studies from India as well as abroad.^7 11 18^ With regards to willingness to participate in a future CR, those who have heard about it, know someone or themselves who have participated in a CR are more likely to enrol in a CR. This could be explained by the psychological phenomenon call mere exposure effect wherein people familiar with the concept involve themselves more.^19^ However, those who exactly know what a CR is seen to be significantly less likely to participate in CR. This could be attributed to the recent infodemic, which probably made people perceive that they know a lot more in detail about CR and that there are issues.

Regarding SES, when we compared low SES (class 5) with all the other classes, it became evident that all the other classes exhibited a tendency towards reduced odds of participating in CR, and class 2, in particular, reached statistical significance. This shows that the higher SES class people do not prefer participating in CR although they show a trend towards willingness to participate in future CR. This raises ethical concerns if the burden of research is not equally distributed across the various socioeconomic classes. This is also in contrast to the developed countries where low SES/ income groups do not participate in CR due to their comparatively lesser access to healthcare.^20^ In India, it is worth noting that participation in CR may often be viewed as an additional income source.^21^ This perspective may have influenced individuals from lower and middle-income groups, who were significantly impacted by the pandemic and subsequent lockdown economically and financially. In contrast, professionals, many of whom were healthcare or frontline workers, although showed a positive inclination towards CR participation, likely due to their higher level of education^22^ were actively engaged in their crucial roles in combating the pandemic, which limited their ability to participate in CR activities. Nevertheless, the Government of India of late has brought in new rules and guidelines governing CR^23^ and it is more likely that these inequalities in participation cease to exist in the near future by coordinated efforts of all stakeholders in increasing awareness among the general public.

The strengths of the current study are that it has a large sample size, including adults from all over India, with a high response rate of 92.8%, which enhances the representativeness of the findings among the college students in India. The survey instrument used was also a validated scale. The limitations of this study are that the design is a cross-sectional survey, which has inherent disadvantages such as differences in interpretation of questions/answer options, extent of understanding the questionnaire and likewise. However, the large sample size is likely to have mitigated this bias. Additionally, the study was limited to students enrolled in an educational institution, potentially affecting the generalisability of the findings to a broader adult Indian population. Similarly, the number of respondents from some of the states and union territories were less as there were challenges to approach those indigenous people due to logistical reasons like hilly terrain, poor connectivity and likewise, potentially making the results less generalisable to the general public of those states/union territories. However, the results are expected to be generalisable to the broad regions of the nation namely east, west, north, south, central and north-east India.

## Conclusion

This study sheds light on the current knowledge and perceptions about CR among young college going adults from non-science backgrounds in India. Despite wide media coverage on the drug development and approval processes during the COVID-19 pandemic, there were notable gaps in knowledge and concerns about various aspects of clinical trials, although some students exhibited positive attitudes towards CR and recognised its societal benefits. These findings suggest a need for targeted educational initiatives and awareness campaigns to improve understanding and trust in CR among the lay public, especially the young adults who form the future generations. Addressing these issues is crucial to fostering a well-informed and actively participating public, which is essential for the growth and success of the clinical trial industry in India and the advancement of medical science. This study also serves as a valuable baseline for future research and gauges the effectiveness of initiatives aimed at bridging these knowledge gaps and enhancing favourable perceptions of CR in India.

## supplementary material

10.1136/bmjph-2023-000748 online supplemental file 1

## Data Availability

Data are available upon reasonable request.
