# Supplementary material for: Knowledge and perceptions about clinical research and its ethical conduct among college students from non-science background: a representative nation-wide survey from India
Source: BMJ Public Health. 2024 May 30;2(1):e000748. doi: 10.1136/bmjph-2023-000748 (PMC11812855; doi:10.1136/bmjph-2023-000748)
Supplement: online supplemental file 1 [file bmjph-2-1-s001.pdf]

Supplementary Table - 1: Perceptions of clinical research among study participants

	Category	No. (%)
If you decide not to participate in research your doctor will not give you good care	False	2861 (59.7)
	True	236 (4.9)
	Not aware	1157 (24.1)
	Not relevant	542 (11.3)
Confidentiality is a matter of importance to research participants	False	151 (3.1)
	True	4004 (83.5)
	Not aware	532 (11.1)
	Not relevant	109 (2.3)
Confidentiality of research participants is adequately protected	False	277 (5.8)
	True	2308 (48.1)
	Not aware	2104 (43.9)
	Not relevant	107 (2.2)
Clinical research information provided by pharmaceutical companies can be trusted	False	899 (18.7)
	True	1461 (30.5)
	Not aware	2263 (47.2)
	Not relevant	173 (3.6)
Clinical research information provided by academic institutions can be trusted	False	597 (12.4)
	True	1874 (39.1)
	Not aware	2154 (44.9)
	Not relevant	171 (3.6)
The most important reason for developing new treatments is financial gain	False	2297 (47.9)
	True	1045 (21.8)
	Not aware	1148 (23.9)
	Not relevant	306 (6.4)
Clinical research benefits society	False	103 (2.1)
	True	3887 (81)
	Not aware	702 (14.6)
	Not relevant	104 (2.2)
Volunteers in clinical research get adequate compensation for their participation	False	750 (15.6)
	True	961 (20)
	Not aware	2978 (62.1)
	Not relevant	107 (2.2)
Clinical research is an essential step in developing new treatments	False	120 (2.5)
	True	3851 (80.3)
	Not aware	737 (15.4)
	Not relevant	88 (1.8)

Human participants in clinical research are treated like experimental animals (as "human Guinea Pigs")	False	1203 (25.1)
	True	1114 (23.2)
	Not aware	2315 (48.3)
	Not relevant	164 (3.4)
The most important reason for developing new treatments is the advancement of science	False	439 (9.2)
	True	3338 (69.6)
	Not aware	894 (18.6)
	Not relevant	125 (2.6)
Altruism is the only valid reason for participation in research	False	1739 (36.3)
	True	617 (12.9)
	Not aware	2201 (45.9)
	Not relevant	239 (5)
Volunteers in clinical research get adequate information about the research they participate in	False	788 (16.4)
	True	1836 (38.3)
	Not aware	2063 (43)
	Not relevant	109 (2.3)
Participation in research is entirely voluntary	False	474 (9.9)
	True	3213 (67)
	Not aware	1002 (20.9)
	Not relevant	107 (2.2)
Participants in clinical research get adequate compensation for any adverse outcomes	False	752 (15.7)
	True	1231 (25.7)
	Not aware	2703 (56.4)
	Not relevant	110 (2.3)
Clinical research harms society	False	2809 (58.6)
	True	289 (6)
	Not aware	1485 (31)
	Not relevant	213 (4.4)
The government always adequately protects the public against unethical clinical research	False	975 (20.3)
	True	1578 (32.9)
	Not aware	2111 (44)
	Not relevant	132 (2.8)
Hospitals that participate in clinical research provide better healthcare	False	548 (11.4)
	True	1570 (32.7)
	Not aware	2355 (49.1)
	Not relevant	323 (6.7)
All the results of clinical research are made available to the public	False	1812 (37.8)
	True	725 (15.1)

	Not aware	2130 (44.4)
	Not relevant	129 (2.7)
Doctors force their patients to participate in research	False	2492 (52)
	True	329 (6.9)
	Not aware	1836 (38.3)
	Not relevant	139 (2.9)
You have had an opportunity to participate in clinical research	False	3028 (63.1)
	True	646 (13.5)
	Not aware	835 (17.4)
	Not relevant	287 (6)
Researchers make sure research is safe for participants	False	479 (10)
	True	2158 (45)
	Not aware	2044 (42.6)
	Not relevant	115 (2.4)
The media accurately describes clinical research	False	1902 (39.7)
	True	609 (12.7)
	Not aware	2089 (43.6)
	Not relevant	196 (4.1)
Experiments on humans are essential to developing new treatments	False	661 (13.8)
	True	2428 (50.6)
	Not aware	1483 (30.9)
	Not relevant	224 (4.7)
Harmful events occurring during a clinical trial must be due to experimental treatment	False	529 (11)
	True	2035 (42.4)
	Not aware	2074 (43.2)
	Not relevant	158 (3.3)
The public should be involved in clinical research (eg: design, oversight and funding)	False	560 (11.7)
	True	2512 (52.4)
	Not aware	1434 (29.9)
	Not relevant	290 (6)
What is the impact on clinical research for collaborations with non-Indian Industry partners?	Bad	218 (4.5)
	Good	2179 (45.4)
	None	237 (4.9)
	Not aware	2162 (45.1)
The extent of the impact on clinical research for collaborations with non-Indian Industry partners	Not responded	2399 (50)
	Large	832 (17.3)
	Minimal	97 (2)
	Moderate	1468 (30.6)

What is the impact on clinical research for collaborations with non-Indian academic partners?	Bad	236 (4.9)
	Good	1752 (36.5)
	None	322 (6.7)
	Not aware	2486 (51.8)
The extent of the impact on clinical research for collaborations with non-Indian academic partners	Not responded	2808 (58.5)
	Large	766 (16)
	Minimal	126 (2.6)
	Moderate	1096 (22.9)
